# The Potential of microRNAs for Stem Cell-based Therapy for Degenerative Skeletal Diseases

**DOI:** 10.1007/s40610-017-0076-4

**Published:** 2017-10-23

**Authors:** Emma Budd, Shona Waddell, María C. de Andrés, Richard O. C. Oreffo

**Affiliations:** Bone & Joint Research Group, Centre for Human Development, Stem Cells and Regeneration, Faculty of Medicine, University of Southampton, Southampton, SO16 6YD UK

**Keywords:** miRNA, Skeletal stem cell, Cartilage, Bone, Osteoarthritis, Osteoporosis

## Abstract

**Purpose of Review:**

Degenerative skeletal disorders including osteoarthritis (OA) and osteoporosis (OP) are the result of attenuation of tissue regeneration and lead to painful conditions with limited treatment options. Preventative measures to limit the onset of OA and OP remain a significant unmet clinical need. MicroRNAs (miRNAs) are known to be involved in the differentiation of stem cells, and in combination with stem cell therapy could induce skeletal regeneration and potentially prevent OA and OP onset.

**Recent Findings:**

The combination of stem cells and miRNA has been successful at regenerating the bone and cartilage in vivo. MiRNAs, including miR-146b known to be involved in chondrogenic differentiation, could provide innovative targets for stem cell-based therapy, for the repair of articular cartilage defects forestalling the onset of OA or in the generation of a stem cell-based therapy for OP.

**Summary:**

This review discusses the combination of skeletal stem cells (SSCs) and candidate miRNAs for application in a cell-based therapy approach for skeletal regenerative medicine.

## Introduction

Degenerative skeletal disorders are specific diseases associated with the ageing bone. These disorders can be divided into two main diseases: osteoarthritis (OA) and osteoporosis (OP). Given OA and OP are the result of the loss of the regenerative capacity of the skeletal tissues, skeletal stem cells (SSCs) have been investigated with the aim of harnessing their potential to improve the symptoms and treatment of these pathologies.

### Ageing Cartilage and the Onset of OA

OA is a prevalent chronic disease and can be described as a heterogeneous condition, which results in joint signs and symptoms associated with defective integrity of the articular cartilage and changes to the bone at joint margins [1]. Articular cartilage is composed of non-migratory and non-proliferative resident chondrocytes embedded within an avascular, alymphatic and aneural specialised extracellular matrix (ECM), factors which following injury are likely to account for the limited capacity of articular cartilage to intrinsically repair [2]. Articular cartilage injury is likely to be causative to the onset of OA. Damage to the articular cartilage may appear asymptomatic but it is extremely likely that over time degenerative changes will result. Messner et al. demonstrated that athletes with isolated chondral lesions did not require treatment following initial injury. However, 14 years later, some of the athletes displayed with a reduction of the joint space, indicating that despite the initial chondral lesions having been asymptomatic, degradation of the articular cartilage supervened leading to permanent knee damage [3]. Cartilage damage is typically succeeded with long-term articular cartilage deterioration and OA.

Articular cartilage deterioration and onset of OA could potentially be prevented by repair of the initial articular cartilage defect. A number of research groups have looked to identify the presence of chondroprogenitor cells within the articular cartilage and in tissues directly surrounding the articular cartilage, such as the synovium [4], the groove of Ranvier [5], the intrapatellar fat pad [6, 7] and the articular cartilage superficial zone [8, 9]. However, the source of progenitor populations for articular cartilage repair needs to be readily accessible and must not induce damage to the articular cartilage or tissues during isolation. Harnessing SSCs from bone marrow offers an option which does not involve further damage directly to articular cartilage or any surrounding tissue. The ability to direct bone marrow-derived SSCs to differentiate towards the chondrogenic lineage is a propitious option for articular cartilage regeneration. Thus, exploitation of mechanisms which govern chondrogenic differentiation of human SSCs could have significant implications for methods to induce novel articular cartilage formation and, potentially, help to prevent OA.

### Loss of Regenerative Capacity of the Bone and Development of OP

The human skeleton reaches peak bone mass at around 30 years of age and, thereafter, bone mass is gradually lost. OP is a degenerative skeletal disorder, characterised by low bone mass and generalised disorder of the bone microarchitecture. OP is observed in men and women (in postmenopausal women, exacerbated by a fall in oestrogen production) and is a common cause of loss of bone mass and subsequent fracture [10]. It is estimated that 70% of inpatient fractures are a consequence of OP [11]. The regenerative capacity of the bone is reduced with age, leading to a decrease in bone mass [12–14]. Bone remodelling and therefore the regenerative potential of the bone is controlled by a careful balance between bone resorption, by osteoclasts, and bone deposition by osteoblasts. In OP, this process of bone remodelling is unbalanced with bone resorption exceeding bone formation resulting in the loss of bone mass observed in OP. The loss of regenerative capacity of the bone is multifactorial including (i) reduced stem cell potency/number, (ii) increased osteoclastic bone resorption, (iii) metabolic/factor imbalance and (iv) reduced osteoblast function [14, 15]. In addition, the increase in bone marrow adiposity is believed to play an important role in OP, with osteoporotic patients exhibiting a higher ratio of adipose tissue to total tissue volume in iliac crest bone biopsies compared to healthy controls [16, 17].

Currently, OP is treated with drugs which aim to increase bone density or inhibit bone resorption. Strategies include the use of bisphosphonates [18, 19], selective oestrogen receptor modulators [20], calcitonin [21, 22], sodium ranelate [23], RANK ligand inhibitors [24], the recombinant form of parathyroid hormone, teriparatide [25] and, more recently, the anti-sclerostin antibody, blosozumab [26, 27]. Although these drugs offer significant treatment options, development of efficacious anabolics for an increasing ageing population remains a goal.

### Potential for Stem Cells and microRNAs for Treatment of Skeletal Disorders

Stem cells have been shown to be regulated in part by microRNAs (miRNAs), which regulate genes involved with differentiation post-transcriptionally. MiRNAs are processed from longer primary transcripts which undergo processing in the nucleus and the cytoplasm to form small non-coding RNA, which average 22 nucleotides in length [28]. Sequence complementarity between a miRNA and its target mRNA determines whether the miRNA induces post-transcriptional inhibition or degradation of the mRNA, which in turn prevents translation and protein synthesis [28]. This ability of miRNAs to regulate translation can allow for the potential exploitation of the function of miRNAs for use to control cellular processes including differentiation. Several miRNAs have been identified to play roles in chondrogenesis and osteogenesis [29–31]. MiRNAs found to be involved in these highly regulated processes, could therefore be exploited for their use to either induce stem cell chondrogenic differentiation for articular cartilage regeneration or osteogenic differentiation for bone regeneration. In essence, stem cells could be utilised for the regeneration of skeletal tissues in concert with miRNAs to enhance the differentiation of transplanted stem cells towards the chondrogenic or osteogenic lineages. Use of miRNAs could prime transplanted stem cells, directing them towards the desired cell fate. MiRNA modulation could serve as a tool to enhance stem cell differentiation, a novel approach to articular cartilage tissue reparation and bone regeneration. Not only could this novel concept induce the regeneration of skeletal tissues but, if applied early enough, could prevent the onset and progression of OA and OP. This review will examine the use of stem cells to regenerate skeletal tissue and the discovery of miRNAs which are involved in the chondrogenic and osteogenic differentiation of stem cells, including our own observations. Examples of studies which have demonstrated the use of miRNA modulated stem cell transplantation in vivo are discussed to reinforce the potential of miRNAs to direct stem cells to regenerate skeletal tissues.

## The Use of Stem Cells for the Treatment of Degenerative Skeletal Disease

### Properties of Skeletal Stem Cells

A stem cell is characterised by its ability to self-renew by means of asymmetrical cell division and its potential to differentiate into specialised types of cells, thereby retaining a pool of stem cells and simultaneously producing transit amplifying cells [32]. Adult stem cells replace degenerating cells which facilitates tissue homeostasis. Adult stem cells can therefore be defined as the regenerators that follow the degeneration process which may occur due to trauma, age or pathogenic conditions [32].

The term skeletal stem cell (SSC), preferred by the authors and used in this review in reference to our own data defines, specifically, a self-renewing stem cell that resides in postnatal bone marrow and can differentiate into cartilage, the bone, haematopoiesis-supportive stroma and marrow adipocytes. It is the SSC of the bone marrow stroma that is responsible for the regenerative capacity inherent to the bone.

The heterogeneous population of cultured plastic adherent cells isolated from the bone marrow should be referred to as bone marrow stromal cells (BMSCs). However, it is acknowledged, the term mesenchymal stem cell (MSC), originally in reference to a hypothetical common progenitor of a wide range of “mesenchymal” (non-haematopoietic, non-epithelial, mesodermal) tissues, is commonly used and in this review will be retained where cited/used by others in the field.

Additional to their differentiation and proliferative properties, SSCs have been proposed to possess immuno-modulatory properties which can regulate tumour evasion, autoimmunity and regulation of transplantation tolerance [33]. A combination of regulatory mechanisms exist within SSCs which act upon several immune cells including dendritic cells, T lymphocytes and natural killer (NK) cells [34]. Tse et al. observed that SSCs failed to stimulate allogeneic peripheral blood mononuclear cells and T cell proliferation and actively inhibited T cell proliferation [35]. Le Blanc et al. showed that alloreactive lymphocyte proliferative responses were not elicited in undifferentiated and also osteogenic and chondrogenic differentiated SSCs [36]. The immunosuppressive properties of SSCs, theoretically, limit any rejection of SSCs that could occur during therapeutic cell transplant. The concept that fibroblast-like cells migrate to distal sites of injury was fist hypothesised by the German pathologist Cohnheim [37]. Stem cells have the potential to home to sites of injury where they are likely to induce repair, through direct differentiation to replace damaged cells and/or secretion of mediators, which creates a reparative environment with immuno-regulatory function and anti-apoptotic regulation [38].

### Therapeutic Potential of SSCs in Degenerative Skeletal Disease

#### Osteoarthritis

Loss of chondrocytes and diminishment of the surrounding specialised ECM is as a result of the inability for cartilage to undergo spontaneous endogenous regeneration. The use of cell-based therapies to repair articular cartilage defects aims to produce a fully functional joint surface, capable of tolerating stress and strain.

Several studies have investigated the potential of SSCs in regenerating cartilage in animal models. For example, Im et al. induced osteochondral defects in to the patella grooves of rabbits, and autologous bone marrow-derived MSCs were applied to the defect sites. Histological and molecular analysis concluded that implantation of cultured MSCs could enhance cartilage repair [39]. In experimentally induced OA joint studies, non-operative administration of MSCs has also shown beneficial effects [40, 41]. A reduction in the degeneration of articular cartilage was observed following injection of autologous bone marrow-derived MSCs, in a hyaluronan solution, directly into OA-induced caprine knee joints [40].

A popular choice amongst research groups for investigating articular cartilage regeneration has been transplantation of SSCs combined with a scaffold. Previously, osteochondral progenitor cells expanded in vitro and dispersed into a type-1 collagen gel were transplanted into a full-thickness surgically induced articular cartilage defect in rabbits. At 24 weeks, the post-implantation subchondral bone was completely repaired with overlying articular cartilage [42]. Furthermore, Berninger et al. have suggested an experimental technique for combining MSCs in fibrin clots, followed by transplantation of pre-established fibrin-cell-clots into osteochondral defects in lapine knee joints. Preliminary experiments observed an intact and homogenous surface 12 weeks following implantation of the fibrin-MSC-clot into defect sites [43].

Previous clinical studies have reported the therapeutic effect of MSCs administration in patients [44–48]. Nejadnik et al. found that patients administered with bone marrow stem cells into chondral lesions demonstrated enhanced physical chondrocyte implantation [44]. Follow-up inspection found that transplantation of autologous expanded bone marrow-derived MSCs combined with platelet-rich fibrin glue, to full-thickness cartilage defects in five patients, resulted in improvement to symptoms in all patients. Complete defect filling and surface conformity with native cartilage was observed in three patients [45]. Kuroda et al. showed that administration of autologous bone marrow stromal cells to an articular cartilage defect in a young male athlete resulted in marked improved clinical outcomes. At 7 months post-surgery, arthroscopy revealed that the defect was completely covered with smooth tissues, and histologically the defect was filled with hyaline-like cartilage. Strikingly, 1 year post-surgery, the athlete returned to his previous activity level and experienced no pain with significant improvement in clinical symptoms [48].

#### Osteoporosis

Given OP is the result of altered bone remodelling, improving the efficiency or restoration of appropriate balance of this process would appear a natural strategy for the treatment of OP. It is known that SSCs can be induced to form osteoblasts when cultured on tissue culture plastic [49]. However, translation to a cell-based treatment requires careful control of the differentiation of the stem cells. This could, potentially, be achieved through the use of miRNAs to control osteogenic differentiation. In addition, ensuring maximal osteogenic differentiation, with minimal differentiation to other lineages, remains a key challenge in translating skeletal stem and progenitor populations from the bench to clinical application. Various strategies have been proposed which would ensure maximal osteogenic lineage commitment. These approaches include selection of a specific stage of osteoprogenitor subsets [50, 51]. Other approaches to select for osteoprogenitor cells include the use of biomaterials to culture SSCs designed to enhance osteogenic differentiation. Examples of biomaterials include nanosurface geometries [52, 53] and osteoconductive scaffolds [54].

The use of bone tissue, autograft (patient derived) and allograft (donor), together with bone stem cells and progenitors has been examined. Marcacci et al., in a study of four patients with large bone defects, examined the potential of autologous culture-expanded SSCs onto a ceramic scaffold [55]. No major complications were reported after surgery and long-term follow-up of 6 to 7 years showed good integration of the scaffold [55]. Kim et al. studied the effect of osteoblast injection into long bone fractures to examine accelerated healing [56]. Autologous osteoblasts were expanded from patients with long bone fracture, and injected into the site of fracture, with the control group receiving no treatment [56]. The results demonstrated that osteoblast injection enhanced fracture healing with little complication [56]. The success of these important, albeit small, trials in humans emphasise the potential of SSC strategies for the treatment of bone fracture, bone defects and potentially degenerative bone diseases. In particular, culturing SSCs with a high osteogenic differentiation potential would prove important to generate the cell numbers required for cell-based therapy [57].

## MiRNA Expression During Skeletal Differentiation of Stem Cells

**Fig. 1:**
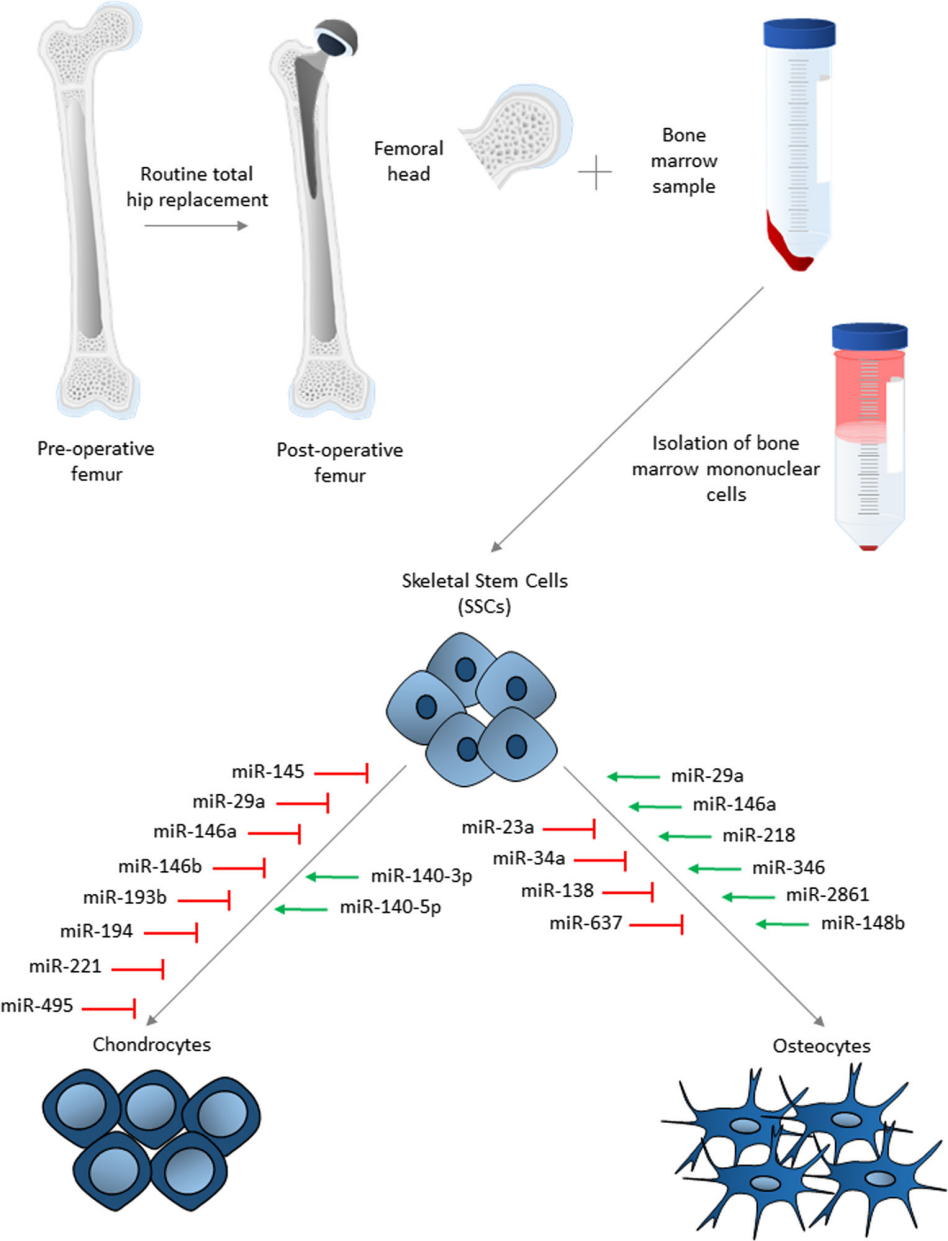
miRNAs involved in osteogenic and chondrogenic differentiation of SSCs isolated from human bone marrow. Following routine total hip replacement, the femoral head is removed and bone marrow sample donated for isolation of SSCs. From the bone marrow sample, mononuclear cells are isolated by density centrifugation and the cell population enriched for SSCs by magnetic separation. MiRNAs involved in either chondrogenic or osteogenic differentiation are indicated by association with the relevant arrow. MiRNAs in red negatively regulate differentiation and in green positively regulate differentiation

In vitro models of stem cell differentiation have allowed for the analysis of miRNAs involved with post-transcriptional regulation of chondrocyte and cartilage development, as well as osteocyte and bone development. Such miRNAs are responsible for gene activation or suppression during the process of differentiation. A selection of miRNAs and their mRNA targets studied to date, known to be involved in stem cell differentiation, are listed in Table 1 (chondrogenic differentiation) and Table 2 (osteogenic differentiation) and further illustrated in Fig. 1. Comprehension of miRNA expression profiles and the role that miRNAs play in regulation of gene expression during differentiation of stem cells allows for a better understanding of molecular mechanisms which regulate stem cell differentiation. Critically, miRNAs which influence stem cell fate could be exploited to induce and enhance stem cells, providing a novel cell-based therapy approach. MiRNAs could induce and enhance transplanted stem cells at articular cartilage defect sites to regenerate articular cartilage. MiRNAs could induce and enhance stem cell osteogenic differentiation generating a cell-based therapy for OP treatment. Through the application of miRNA mimics or miRNA inhibitors, stem cell differentiation can be modulated to enhance direction towards the desired lineage. Tables 1 and 2 indicate potential miRNAs of which expression levels could be increased or decreased, using miRNA mimics and miRNA inhibitors, respectively, which could potentially enhance chondrogenic and osteogenic differentiation.

**Table 1:** MiRNAs identified in chondrogenic differentiation of stem cells, target mRNA, effect of miRNA modulation on chondrogenesis and potential use of identified miRNAs to enhance chondrogenic differentiation

MiRNA	Expression during chondrogenesis	mRNA targets of miRNA and mRNA function in chondrogenesis	Reported effect of miRNA modulation on chondrogenesis	Potential use of miRNA in inducing chondrogenesis
miR-29a	miR-29a was reported to be down-regulated during chondrogenic differentiation of human MSCs (hMSCs) [58, 59].	miR-29a was demonstrated to directly target the 3’UTR of *FOXO3A*. Transcription factor *FOXO3A* was observed to be up-regulated during chondrogenesis, and modulation of *FOXO3A* expression was found to regulate *SOX9*, *AGCAN* and *COL2A1* expression, with FOXO3A binding sites also identified within the genomic sequences of these genes [59]. miR-29a is likely to be down-regulated during chondrogenesis enabling derepression of *FOX3A* expression.	The overexpression of miR-29a in hMSCs, using pre-miR-29a, resulted in inhibition of chondrocyte-specific markers and a suppressive effect on chondrogenic differentiation. [59].	Decrease endogenous miR-29a levels with a miR-29a inhibitor.
miR-140-3p	miR-140-3p was reported to be up-regulated during chondrogenic differentiation of hMSCs [60].	mRNA targets of miR-140-3p remain unknown [60].	–	Increase miR-140-3p levels with a miR-140-3p mimic.
miR-140-5p	miR-140-5p was reported to be up-regulated during chondrogenesis of hMSCs [60, 61] and equine cord blood mesenchymal stromal cells [62].	MiR-140-5p was demonstrated to directly target the 3’UTR of *RALA*. *RALA* encodes Ras-related protein Ral-A (RALA), a small GTPase which functions to bind and hydrolyse guanosine triphosphate. RALA has been shown to interact with the exocyst complex in mediating cytoskeletal and secretory pathways [63]. RALA has been shown to be involved in TGF-β signalling through internalisation of membrane receptor activin type II [64] and also may be involved with the trafficking and secretion of glycosaminoglycans [65].	Inhibition of endogenous miR-140-5p in differentiating hMSCs, using anti-miR-140-5p, resulted in impaired chondrogenesis with an observed down-regulation of SOX9 and aggrecan. Knockdown of RALA resulted in the up-regulation of SOX9 [60].	Increase miR-140-5p levels with a miR-140-5p mimic.
miR-145	miR-145 was reported to be down-regulated during chondrogenic differentiation of murine MSCs [66].	miR-145 was demonstrated to directly target the 3’UTR of *Sox9* [66]. SOX9 is required for aggrecan [67], Col2a1 [68], Col9a1 [69] and Col11a2 expression [70] and has been shown to bind to chondrocyte-specific enhancer elements in all of these genes. miR-145 is likely to be down-regulated during chondrogenesis enabling derepression of *Sox9* expression.	Overexpression of miR-145 in C3H10T1/2 cells, using pre-miR-146, resulted in the down-regulation of chondrogenic differentiation, evidenced by down-regulation of Sox9 protein and *Col2a1*, *Agcan*, *Col9a2*, *Col11a1* and *COMP* mRNA. Endogenous inhibition of miR-145, using anti-miR-145, resulted in enhancement of chondrogenic differentiation, evidenced by the up-regulation of Sox9 protein and *Col2a1*, *Agcan*, *Col9a2*, *Col11a1* and *COMP* mRNA [66].	Decrease endogenous miR-145 levels with a miR-145 inhibitor.
miR-146a	miR-146a was reported to be down-regulated in chondrogenic epiphyseal cell populations isolated from the epiphyses of human foetal femora [71].	miR-146a was suggested to target positive mediators of chondrogenic signalling, *SMAD2* and *SMAD3* [71]. miR-146a is likely to be down-regulated during chondrogenesis enabling derepression of *SMAD2* and *SMAD3* expression.	Overexpression of miR-146a in cells derived from the epiphyses of human foetal femora, using miR-146a mimic, resulted in *SOX9* down-regulation, indicating the negative effect of miR-146a on chondrogenesis [71].	Decrease endogenous miR-146a levels with a miR-146a inhibitor.
miR-146b	miR-146b was reported to be down-regulated during chondrogenic differentiation of human SSCs [72•].	miR-146b was suggested to target early chondrogenic transcription factor *SOX5* [72•]. Early transcription factor SOX5 is co-expressed with SOX6 and SOX9 to enhance *Col2a1* expression [73] and to enable SOX9 binding to the *AGCAN* enhancer [67]. miR-146b is likely to be down-regulated during chondrogenesis, enabling derepression of *SOX5* expression.	Overexpression of miR-146b in human SSCs, using miR-146b mimic, resulted in down-regulation of SOX5 [72•].	Decrease endogenous miR-146b levels with a miR-146b inhibitor.
miR-193b	miR-193b was reported to be up-regulated during chondrogenic differentiation of human adipose-derived stem cells (hADSCs) [74, 75] and ATDC5 cells [74].	miR-193b was demonstrated to directly target the 3’UTRs of *Tgfb2* and *Tgfbr3* [74].	Overexpression of miR-193b in ATDC cells, using miR-193b mimic, resulted in the down-regulation of chondrogenic differentiation, evidenced by the down-regulation of early chondrogenic markers *col2a1*, *sox9* and *comp* as well as *Tgfb2* and *Tgfbr3*. Inhibition of endogenous miR-193b, using anti-miR-193b, resulted in the enhancement of chondrogenic differentiation, evidenced by the up-regulation of the early chondrogenic markers and *Tgfb2* and *Tgfbr3* [74].	Decrease endogenous miR-193b levels with a miR-193b inhibitor.
miR-194	miR-194 was reported to be down-regulated during chondrogenic differentiation of hADSCs [76].	miR-194 was demonstrated to directly target the 3’UTR of *SOX5* [76]. Early transcription factor SOX5 is co-expressed with SOX6 and SOX9 to enhance *Col2a1* expression [73] and to enable SOX9 binding to the *AGCAN* enhancer [67]. miR-194 is likely to be down-regulated during chondrogenic differentiation enabling derepression of *SOX5* expression.	Overexpression of miR-194 in hADSCs, using pre-miR-194, resulted in the down-regulation of chondrogenic differentiation, evidenced by the down-regulation of the chondrogenic markers *COL2A1*, *COL9A2*, *COL11A1*, *AGC1* and *COMP*. Inhibition of endogenous miR-194, using anti-miR-194, resulted in enhanced chondrogenesis evidenced by up-regulation of chondrogenic markers [76].	Decrease endogenous miR-194 levels with a miR-194 inhibitor.
miR-221	miR-221 was reported to be up-regulated during JNK inhibitor-induced chondrogenic differentiation inhibition in chick limb bud mesenchymal cells [77].	miR-221 was demonstrated to directly target *MDM2* [77].	Silencing of miR-211 in hMSCs resulted in the up-regulation of chondrogenic markers such as *COL2A1* and *SOX9* [78]. In an in vivo cartilage defect model, miR-221 silenced and alginate encapsulated hMSCs, generated cartilaginous tissue with enhanced cartilage repair [79••].	Decrease endogenous miR-221 levels with a miR-221 inhibitor.
miR-495	miR-495 was reported to be down-regulated during chondrogenic differentiation of hMSCs [80•].	miR-495 was demonstrated to directly target the 3’UTR of *SOX9* [80•]. SOX9 is required for aggrecan [67], Col2a1 [68], Col9a1 [69] and Col11a2 expression [70] and binds to chondrocyte-specific enhancer elements in all of these genes. miR-495 is likely to be down-regulated during chondrogenesis enabling derepression of *SOX9* expression.	Overexpression of miR-495 in hMSCs during chondrogenic differentiation, using miR-495 mimic, resulted in the down-regulation chondrogenic differentiation, evidenced by down-regulation of *SOX9*, *COL2A1* and *AGCAN* mRNA. Inhibition of endogenous miR-495, using anti-miR-495, resulted in the enhancement of chondrogenic differentiation, evidenced by up-regulation of *SOX9*, *COL2A1* and *AGCAN* mRNA [80•].	Decrease endogenous miR-495 levels with a miR-495 inhibitor.

**Table 2:** MiRNAs identified in osteogenic differentiation of stem cells, target mRNA, effect of miRNA modulation on osteogenesis and potential use of identified miRNAs to enhance osteogenic differentiation

MiRNA	Expression during osteogenesis	mRNA targets of miRNA and mRNA function in osteogenesis	Reported effect of miRNA modulation on osteogenesis	Potential use of miRNA in inducing osteogenesis
miR-23a	miR-23a was reported to be down-regulated during osteogenic differentiation of human BMSCs (hBMSCs) [81].	miR-23a was demonstrated to directly target the 3’UTR of *LRP5*; an essential component of the Wnt signalling pathway. miR-23a is likely to be down-regulated during osteogenesis, enabling derepression of *LRP5* expression, subsequently enabling Wnt signalling to direct osteogenesis [81].	Overexpression of miR-23a, using miR-23a mimic, resulted in the down-regulation of osteogenic differentiation, evidenced by the down-regulation of *ALP*, *OPN*, *RUNX2* and *IBSP* mRNA. Inhibition of endogenous miR-23a, using anti-miR-23a, resulted in enhancement of osteogenic differentiation, evidenced by the up-regulation of *ALP*, *OPN*, *RUNX2* and *IBSP* mRNA [81].	Decrease endogenous miR-23a levels with a miR-23a inhibitor.
miR-29a	miR-29a was reported to be up-regulated during the osteogenic differentiation of hFOB1.19 cells [82].	miR-29a was demonstrated to directly target the 3’UTRs of negative regulators of Wnt signalling: *Dkk1*, *Kremen2* and *sFRP2* [82]. miR-29a is likely to be up-regulated during osteogenesis to inhibit the negative regulators of Wnt signalling, indirectly promoting osteogenic differentiation.	Inhibition of endogenous miR-29a during osteogenic differentiation of hFOB1.19 cells, using miR-29a inhibitor, resulted in down-regulation of osteogenic differentiation, evidenced by down-regulation of *OCN* and *ALP* mRNA. Overexpression of miR-29a, using miR-29a mimic, resulted in the up-regulation of *OCN* mRNA [82].	Increase miR-29a levels with a miR-29a mimic.
miR-34a	miR-34a was reported to be up-regulated during osteogenic differentiation of hBMSC; however, miR-34a was found to negatively regulate differentiation [83••].	miR-34a was observed to target *JAG1* and cell cycle regulators *CDK4* and *CDK6* [83••].	Inhibition of endogenous miR-34a, using anti-miR-34a, was found to enhance osteogenic differentiation. hBMSCs transfected with anti-miR34a were subcutaneously implanted in mice, with the support of a scaffold. In this in vivo model of bone regeneration, an increase in bone formation was observed in mice which had received miR-34a silenced hBMSCs [83••].	Decrease endogenous miR-34a levels with a miR-34a inhibitor.
miR-138	miR-138 was reported to be down-regulated during osteogenic differentiation of human MSCs (hMSCs) [84].	miR-138 was demonstrated to directly target the 3’UTR of *PTK2*, which encodes focal adhesion kinase (FAK) [84]. During osteoblast differentiation is it thought that Grb2-Sos-Ras pathway is activated by FAK inducing ERK1/2, and subsequently downstream genes associated with osteogenesis [85].	Inhibition of endogenous miR-138 in hMSCs, using anti-miR-138, resulted in enhanced osteogenic differentiation as measured by an increase in *OCN* and *ALP* mRNA levels and matrix mineralisation. Overexpression of miR-138, using pre-miR-138, was found to reduce osteogenic differentiation. miR-138 silenced hMSCs loaded onto a scaffold and implanted subcutaneously in mice, resulted in an increase in bone formation and up-regulation *OCN* and *ALP* mRNA [84].	Decrease endogenous miR-138 levels with a miR-138 inhibitor.
miR-146a	miR-146a was reported to be up-regulated in osteogenic diaphyseal cell populations isolated from the diaphysis of human foetal femora [72•].	miR-146a targets positive regulators of chondrogenesis, *SMAD2* and *SMAD3*. Indirect promotion of osteogenic differentiation is suggested via miR-146a degradation of *SMAD2* and *SMAD3*, during endochondral foetal skeletogenesis [72•].	miR-146a overexpression, using miR-146a mimic, resulted in down-regulation of SMAD2 and SMAD3 and an increase in *RUNX2* mRNA, a marker of osteogenic differentiation [72•].	Increase miR-146a levels with a miR-146a mimic.
miR-218	miR-218 was reported to be up-regulated in MC3T3 cells [86] and hADSCs [87•] during osteogenic differentiation.	miR-218 was demonstrated to directly target the 3’UTR of negative regulators of Wnt signalling, *SFRP2* and *DKK1* [86].	Overexpression of miR-218 in murine BMSCs, using miR-210 lentivirus, resulted in enhanced osteogenic differentiation, evidenced by up-regulation of *Runx2*, *Alp* and *Ocn* mRNA [81]. Inhibition of miR-218 was found to reduce osteogenic differentiation of hADSCs [87•].	Increase miR-218 levels with a miR-218 mimic.
miR-346	miR-346 was reported to be up-regulated during osteogenic differentiation of hBMSCs [88].	miR-346 was demonstrated to directly target the 3’UTR of *GSK-3β*, a negative regulator of Wnt signalling. An increase in nuclear accumulation of β-catenin was observed during miR-346 overexpression, indicating an enhancement of Wnt signalling. miR-346 was suggested to promote osteogenesis through activation of the Wnt signalling pathway [88]. miR-346 is likely to be up-regulated during osteogenesis to inhibit the negative regulators of Wnt signalling, indirectly promoting osteogenic differentiation.	Overexpression of miR-346 in hBMSCs, using miR-346 mimic, resulted in enhanced osteogenic differentiation, evidenced by up-regulation of *RUNX2*, *ALP* and *OPN* mRNA and increased matrix mineralisation and ALP activity. Inhibition of endogenous miR-346, using anti-miR-346, resulted in the down-regulation osteogenic differentiation, evidenced by down-regulation of osteogenic marker mRNA expression, ALP activity and matrix mineralisation [88].	Increase miR-346 levels with a miR-346 mimic.
miR-637	miR-637 was reported to be down-regulated during osteogenic differentiation of hMSCs [89].	miR-637 was demonstrated to directly target the 3’UTR of *OSX*, which encodes osterix, a key transcription factor of osteoblasts. Down-regulation of miR-637 during osteogenesis results in osterix derepression, promoting osteogenic differentiation [89].	Overexpression of miR-637 in hMSCs, using a lentiviral- pre-miR-673 vector, down-regulated osteogenic differentiation, evidenced by a decrease in ALP activity and down-regulation of *RUNX2* and *BMP2* mRNA. Inhibition of endogenous miR637, using a lentiviral short-hairpin of pre-miR-637 vector, enhanced osteogenic differentiation, evidenced by an increase in ALP activity and up-regulation of *RUNX2* and *BMP2* mRNA [89].	Decrease endogenous miR-637 levels with a miR-637 inhibitor.
miR-2861	miR-2861 was reported to be up-regulated in BMP2-induced osteogenesis of murine BMSCs [90].	miR-2861 was demonstrated to directly target an amino acid coding sequence in *Hdac4* mRNA [90]. miR-2861 is likely to be up-regulated during osteogenesis to suppress expression of the negative regulator of Runx2, *hdac4*.	Overexpression of miR-2861 in mice BMSCs, using pre-miR-2861, resulted in enhanced osteogenic differentiation, evidenced by increased ALP activity and osteocalcin secretion and up-regulation of Runx2. An in vivo model reported that injection of anti-miR-2861 in mice resulted in a reduction in femur bone mineral density and reduced osteoblast activity [90].	Increase miR-2861 levels with a miR-2861 mimic.

## Therapeutic Potential of Modulating miRNAs for Skeletal Disorders

Given that miRNAs display the potential to regulate chondrogenic and osteogenic differentiation of stem cells, harnessing miRNAs offers an appealing strategy for skeletal tissue repair of cartilage or enhancement of differentiation of SSCs towards an osteogenic lineage for bone formation. The potential of miRNAs to augment articular cartilage regeneration has been demonstrated in a study conducted by Lolli and colleagues [79••]. MiR-221 has been identified as a negative regulator of chondrogenesis [77, 78]. Lolli et al. have previously shown that silencing miR-221 induced chondrogenic differentiation of hMSCs [78]. hMSCs transfected with an inhibitor of miR-221 were encapsulated in alginate. A cartilage defect in an osteochondral biopsy was then filled with the transfected and alginate encapsulated cells, followed by implantation of the biopsy into immunocompromised mice. Compared to control untreated hMSCs and alginate only controls, miR-221 silenced hMSCs enhanced cartilage repair in vivo and cartilaginous tissue was generated with no sign of hypertrophic associated type X collagen deposition [79]. This approach, combining hMSCs primed with miRNA inhibitor, in an in vivo cartilage defect model is the first of its kind and suggests a translational strategy to localise stem cells to defective cartilage sites and promote cartilage repair.

The potential of miRNAs to augment bone formation has been demonstrated in a number of murine studies. With both miR-138 and miR34a, a hydroxyapatite/tricalcium phosphate (HA/TCP) scaffold was utilised in order to localise stem cells subcutaneously. Chen et al. used a similar approach to study the role of miR-34a, which is a negative regulator of bone formation [83••]. hMSCs were transfected with pre-miR-34a, anti-miR-34a and control miR and loaded onto HA/TCP scaffolds and implanted subcutaneously into immunocompromised mice. Implantation of the scaffold with hMSCs transfected with anti-miR-34a resulted in a more than 3.5-fold increase in bone formation [83••]. Eskildsen et al. used lipofectamine to transfect pre-miR-138, anti-miR-138 and control miR into hMSCs [84]. The cells were loaded onto HA/TCP scaffold and implanted subcutaneously into immunocompromised mice. Implantation of the scaffold comprising hMSCs transfected with anti-miR-138 resulted in a 2.2-fold increase in bone formation. While, implantation of the scaffold comprising hMSCs transfected with miR-138 mimic resulted in a 6.7-fold decrease in bone formation, supporting the observation that miR-138 is a negative regulator of osteogenic differentiation and bone formation [84]. This approach, combining hMSC primed with miRNA inhibitor or mimic and a scaffold, suggests translational strategies to localise stem cells to the bone.

Li et al. used a miRNA intravenous therapy approach, without the use of the scaffold, to investigate the role of the positive regulator of osteogenic differentiation, miR-2861, on bone formation in mice [90]. When antagomiR miR-2861 was intravenously administered to induce miR-2861 silencing, a decrease in femur mineral density and trabecular thickness was observed. Following on from this work, Li et al. studied the role of miR-2681 in the development of OP in human patients with primary OP. The authors identified in a human sibling pair, both suffering from OP, an undetectable expression level of miR-2861 in their bone. A homozygous single nucleotide polymorphism (SNP) in pre-miR-2861 was identified and was suggested to be accountable for negligible miR-2861 expression levels and likely to be the confounding factor in the pathogenesis of primary OP. The authors suggest that dysregulation of miR-2861 is likely to induce defective osteoblast differentiation and subsequently contribute to OP. This mutation was found to be heterozygous in the parents of the sibling pair and these family members also suffered from OP. However, when extended to a larger cohort of 369 patients, the same SNP in pre-miR-2861 was not identified, indicating that the SNP was uncommon and not reflective of the general osteoporotic population. Nevertheless, the importance of miR-2861 in osteogenic differentiation and OP was highlighted, indicating its potential as a therapeutic approach.

For successful use of miRNA in stem cell therapeutics, it will be important to localise and minimise any miRNA off target effects. Qureshi et al. developed a technique for photoactivation of nanoparticle conjugated miR-148b [91•]; miR-148b has previously been reported to up-regulate osteogenic differentiation, increasing ALP activity in hMSCs [92]. The non-toxic conjugate remained inert until photoactivation by UV light, which was confirmed by an observed increase in *ALP* and *OCN* expression in photoactivated hADSCs compared to non-UV treated cells. In addition, the specific use of nanoparticle conjugated miR-148b resulted in delivery of miR-148b to the intracellular compartments of hADSCs, without the need for additional, potentially damaging, chemical-based methods of transfecting stem cells.

## Conclusion

The problems associated with degenerative skeletal disorders highlighted in this review indicate how miRNA could be used to treat these musculoskeletal conditions. The underlying aetiology of OA remains unknown which makes development of a treatment for this debilitating disease difficult. However, if initial chondral lesions can be targeted, the potential for a preventative approach in OA will become a clinical possibility. If the original chondral lesion can be repaired using stem cells enhanced to undergo chondrogenic differentiation efficiently with use of miRNAs modulation, inducing regeneration of the articular cartilage and reinstating integrity, then the degenerative changes, typical of OA could be reduced. Thus, an attractive approach, with knowledge of different miRNAs expression during chondrogenic differentiation, would be to administer specific miRNAs transfected stem cells to chondral defect sites to enhance articular cartilage regeneration capacity. The bone regeneration balance lost in osteoporosis can benefit from an SSC-based cell therapy which could potentially restore bone microarchitecture and composition to a healthy state. The approach of priming these SSCs with miRNA could lead to enhanced direction of SSCs towards osteogenic differentiation. MiRNAs have been shown to enhance bone formation in murine trials, and known mutations in miRNAs have been identified in human osteoporotic patients. This cell-based approach could be advantageous when applied at early stages of the disease in order to prevent further bone loss and minimise any potential fracture risk that can occur with disease progression. While consideration of miRNAs in skeletal disease therapy is still in its infancy, with considerable research still to be undertaken, the potential for the use of miRNA in a therapeutic context offers an exciting treatment option for a growing ageing population.
